# Circulating biomarkers in osteosarcoma: new translational tools for diagnosis and treatment

**DOI:** 10.18632/oncotarget.19852

**Published:** 2017-08-03

**Authors:** Lavinia Raimondi, Angela De Luca, Viviana Costa, Nicola Amodio, Valeria Carina, Daniele Bellavia, Pierfrancesco Tassone, Stefania Pagani, Milena Fini, Riccardo Alessandro, Gianluca Giavaresi

**Affiliations:** ^1^ Rizzoli Orthopedic Institute, Bologna, Italy; ^2^ Rizzoli Orthopedic Institute, Innovative Technology Platforms for Tissue Engineering, Theranostic and Oncology, Palermo, Italy; ^3^ Department of Experimental and Clinical Medicine, Magna Graecia University of Catanzaro, Catanzaro, Italy; ^4^ Biology and Genetics Unit, Department of Biopathology and Medical Biotechnology, University of Palermo, Palermo, Italy; ^5^ Rizzoli Orthopedic Institute, Laboratory of Preclinical and Surgical Studies, Bologna, Italy; ^6^ Institute of Biomedicine and Molecular Immunology (IBIM), National Research Council, Palermo, Italy

**Keywords:** biomarkers, osteosarcoma, personalized medicine, liquid biopsy, blood serum

## Abstract

Osteosarcoma (OS) is a rare primary malignant bone tumour arising from primitive bone-forming mesenchymal cells, with high incidence in children and young adults, accounting for approximately 60% of all malignant bone tumours. Currently, long-term disease-free survival can be achieved by surgical treatment plus chemotherapy in approximately 60% of patients with localized extremity disease, and in 20–30% of patients with metastatic lung or bone disease. Diagnosis of primary lesions and recurrences is achieved by using radiological investigations and standard tissue biopsy, the latter being costly, painful and hardly repeatable for patients. Therefore, despite some recent advances, novel biomarkers for OS diagnosis, prediction of response to therapy, disease progression and chemoresistance, are urgently needed. Biological fluids such as blood represent a rich source of non-invasive cancer biomarkers, which allow to understand what is really happening inside the tumour, either at diagnosis or during disease progression. In this regard, liquid biopsy potentially represents an alternative and non-invasive method to detect tumour onset, progression and response to therapy. In this review, we will summarize the state of the art in this novel area, illustrating recent studies on OS. Although the data reported in literature seem preliminary, liquid biopsy represents a promising tool with the potential to be rapidly translated in the clinical practice.

## INTRODUCTION

Osteosarcoma (OS) is the most common primary bone cancer, histologically characterized by the production of osteoid by malignant cells; its incidence has a bimodal age distribution, with an initial peak at 12–14 years of age and a second after 60th [1, 2]. OS is a relatively rare malignancy representing approximately 1% of all newly diagnosed cancers in adults, and 3–5% in children [3, 4]. In non metastatic OS, the five years survival rate of patients is between 40% and 75%, due to the introduction of comprehensive treatment including two to three rounds of chemotherapy, followed by definitive resection, and additional adjuvant chemotherapy. Unfortunately, about 20% of patients exhibiting metastases at diagnosis, primarily in the lungs, have a poorer prognosis. Notably, a major issue in the treatment of OS patients is represented by chemotherapy resistance, which can also favour the rapid growth of metastatic lesions [5, 6].

Due to its high chromosomal instability, OS is characterized by extremely complex karyotypes, including various copy number gains and losses [7, 8]. Several studies highlighted genomic alterations in OS cells and suggested potential candidate genes driving OS pathogenesis, although the precise relationship between genetic instability and the development of OS is still under debate [9]. Gains or losses of entire chromosomes or chromosomal segments have been observed in different regions, containing oncogenes, like MYC and COSP3 [8, 10], and tumour suppressor genes, like LSAMP, CDKN2A, RB1, and TP53 [11–13].

Gene mutations, certain bone diseases, inherited cancer syndromes and ionizing radiation represent well-known risk factors for OS development (Table 1); on the other hand, trauma does not seem a major determinant of OS development, but rather a manifestation of the disease when the tumour weakens a bone so much that it breaks (pathological fracture) [14]. In turn, genetic and environmental factors activate a plethora of cancer-related molecular pathways, whose role in OS pathogenesis is nowadays a matter of intense investigation [15–17].

**Table 1:** Risk factors in OS development

**AGE**: OS is more frequent between the ages of 10 and 30, especially during the teenage growth spurt. A link between rapid bone growth and risk of tumour formation has been described.	Reference: [2]
**GENDER**: OS is more common in males than in females	[2]
**INHERITED GENETIC CONDITIONS:** ❖ Autosomal dominant disorder **Li-Fraumeni**, characterized by the germline mutation of the gene p53. ❖ Inherited form of **retinoblastoma**, characterized by the germline mutation in RB1 gene. ❖ Inherited autosomal recessive disorder **Rothmund-Thomson syndrome**, characterized by a mutation in the RECQL4 helicase gene. ❖ Inherited autosomal recessive disorder **Bloom syndrome**. ❖ Rare disorder **Diamond–Blackfan anaemia** characterized by congenital deficiency of red blood cell precursors resulting in pure red cell aplasia.	❖ [159–161] ❖ [7, 162] ❖ [163–164] ❖ [165] ❖ [166]
**PAGET DISEASE**: focal disorder of bone metabolism characterized by abnormal osteoclast resorption activity associated with inadequate remodelling mechanisms.	[160, 167]
**RADIATION THERAPY**: Exposure to ionizing radiation, either inadvertently or high doses of therapeutic radiation, induces DNA damage by conferring distinctive mutational signatures.	[168]
**ALKYLATING AGENTS**: Exposure to alkylating antineoplastic agents, usually used as chemotherapeutics, increases the risk of develop secondary OS, especially in primary Ewing sarcoma patients receiving chemotherapy associated with high doses of radiotherapy.	[169]

The diagnosis of OS includes a set of clinical analyses, radiological investigations and the evaluation of the pathological tissue by performing biopsy. Radiography is routinely used as first line imaging modality for evaluating primary bone tumours. Once suspected diagnosis of OS, magnetic resonance imaging (MRI) is performed in order to understand the distribution of the tumour within the bone, to evaluate the presence of soft tissue masses and to detect skip metastases. Even if computed tomography (CT) scanning is less sensitive than MRI in local evaluation of the tumour, it is recommended to detect lung metastases. Likewise, bone scintigraphy permits to detect osseous metastasis in OS, while the role of positron emission tomography (PET) and integrated PET/CT imaging is increasingly considered by clinicians, particularly for monitoring the effect of chemotherapy or to predict progression-free survival [18–23].

To integrate the diagnostic imaging results, histologic analysis performed on biopsy material provides the definitive diagnosis, giving information on the grade of the tumour (Table 2). Bone biopsy can be performed by a core needle method, under the supervision of an interventional radiologist, guided by radiologic imaging [24]. When the site for bone biopsy is not easily accessible, it is associated with surgery, where the orthopedic surgeon removes the entire tumour by performing an open biopsy with a surgical incision. In localized OS, with the advent of chemotherapy, the complete removal or amputation of the affected limb is reduced to approximately 20% of patients. For metastatic and recurrent OS, following surgical removal of the primary tumour, all operable metastases, most of which in the lungs, need to be removed to significantly improve prognosis. Furthermore, to counteract the possible presence of occult micro-metastasis at diagnosis, routine use of systemic adjuvant chemotherapy increases and improves patient survival [25, 26]. Most of OS patients are treated with chemotherapy before surgery (neoadjuvant chemotherapy), followed by adjuvant chemotherapy, which usually comprises high dose of methotrexate with leucovorin rescue (also known as Folinic Acid, a medication used to decrease the toxic effects of Methotrexate), Doxorubicin, Cisplatin, with or without Ifosfamide. Usually, 2 or more chemotherapeutic agents can be given together, even if the adverse effects reported in multi-agent chemotherapy treatments may result in toxicity [6, 27].

**Table 2:** Two staging system employed for OS

Enneking MSTS staging system
**Stage I** Low-grade, no metastasis IA: intra compartmental IB: extra compartmental **Stage II** High-grade, no metastasis IIA: intra compartmental IIB: extra compartmental **Stage III** Low or High-grade, presence of metastasis

AJCC staging system
**Stage I** Low-grade, no metastasis IA: size < 8 cm IB: size > 8 cm **Stage II** High-grade, no metastasis IIA: size < 8 cm IIB: size > 8 cm **Stage III** Skip metastasis (any grade, any size) **Stage IVA** Pulmonary metastasis (any grade, any size) **Stage IVB** Non Pulmonary metastasis (any grade, any size)

1. Staging system of the Musculoskeletal Tumour Society (MSTS) [170]. 2. Staging system for bone sarcoma of the American Joint Committee on Cancer System (AJCC).

Finally, recent advances in cancer immunology led to reconsider immunotherapy in OS, as recent research has shown that treatments with monoclonal antibodies designed to inhibit immune checkpoint mechanisms (Ipilimumab and Nivolumab) can have therapeutic benefit in patients with OS and lung metastases. Moreover, cytokine therapies seem to be crucial in orchestrating the immune response in OS; for example, administration of IL-2 was able to induce immune activation, probably via NK cells activation, improving prognosis of OS patients [28–31]. The usefulness of laboratory markers, generally represented by alkaline phosphatase (ALP) and lactate dehydrogenase (LDH), is still considered controversial, although high LDH levels are detected in about 40% of OS cases and correlate with adverse prognosis. Several studies investigated serum levels of ALP and LDH, indicating that serum concentrations are not always predictive of response to treatment and/or disease progression [32].

Likewise, bone resorption markers, such as β-isomerized C-terminal telopeptides (β-CTx) and total procollagen type 1 amino-terminal propetide (tP1NP), were found at higher levels in serum of OS patients respect to healthy volunteers. However, β-CTx and tP1NP serum levels were irregular in patients during disease progression, thus not representing valuable candidate biomarkers [33]. Nevertheless, reliable and clinically useful blood markers are currently lacking. On this basis, there is an urgent need for identifying novel and effective biomarkers for OS diagnosis and outcome prediction.

**Figure 1:**
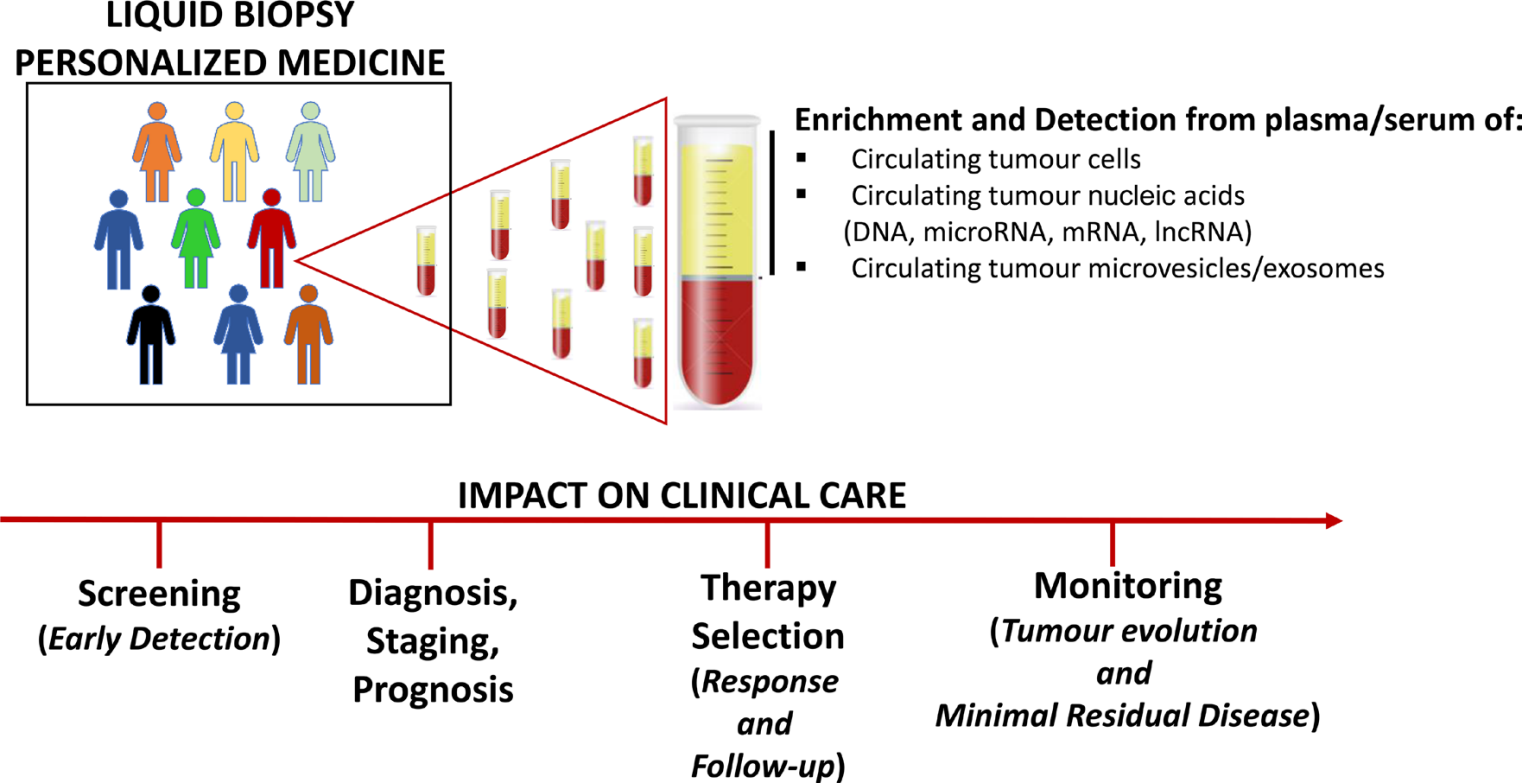
Clinical application of liquid biopsy into the management of cancer

Currently, the analysis of bioptic tissues from primary lesions remains the gold standard to gain essential information on diagnosis, prognosis, prediction of response or resistance to treatment. Tissue biopsy usually provides detailed information on cancer tissue architecture, allowing molecular and histological analysis; unfortunately, such approach has several limitations being an invasive and costly technique, which relies upon few cells or tissue sections that do not necessarily recapitulate the heterogeneity of the whole tumour. Furthermore, multiple biopsies are not feasible for many types of patients, such as the elderly or those with comorbid conditions. In contrast to conventional biopsy, liquid biopsy of tumour components in blood represents a simple and rapid test, easily performed and requiring small amount of sample (usually 10–15 ml of blood), potentially opening new possibilities for cancer characterization and management. It represents a rich source of non invasive biomarkers, useful from early cancer detection to therapy selection and cancer patient monitoring during the course of disease (Figure 1).

An overview of the recent information about candidate circulating biomarkers in OS is reported below.

## LIQUID BIOPSY

Liquid biopsy can be performed to detect circulating tumour microRNAs (miRNAs) and long non-coding RNAs (lncRNAs), circulating tumour cells (CTCs), circulating tumour DNA (ctDNA), and microvesicles/exosomes released into the blood from malignant cells. Such novel approach does not require surgical biopsy, but still provides clinicians with a wide range of information, including tumour origin, staging, patients’ response to therapy and emergence of drug resistance. By performing repeated sampling, liquid biopsy may be useful for early tumour assessment and may improve the identification of micro-metastasis and minimal residual disease, only partially detectable by conventional diagnostic methods. Notably, it may reflect the entire heterogeneity of the disease, useful for better monitoring the molecular changes within the tumour in real time [34].

Liquid biopsy offers remarkable advantages over traditional tissue biopsy, however researchers need to elaborate accurate and reproducible blood tests for routine clinical practice (Table 3). In particular, variables in preanalytical (collection, preservation and storage of biosamples), analytical (quantification and analysis of biosamples) and post-analytical (data collection and interpretation) phases should be considered and validated in depth. Furthermore, liquid biopsy test needs to be more: 1) sensitive, capable to detect precisely small amounts of circulating tumour components, and 2) specific, capable to correctly identify and discriminate among tumour circulating components and non-tumour derived components [35].

**Table 3:** Advantages and limitations of liquid biopsy for selecting a cancer treatment

LIQUID BIOPSIES (circulating tumour cells, circulating nucleic acids, exosomes)		
**ADVANTAGES** ● Isolation from many biological fluids and sources of non-invasive biomarkers ● Less expensive, quick and easily repeatable tests ● Minimal Pain/Risk ● Non-invasive techniques for early cancer diagnosis and monitoring of disease progression (real-time monitoring of patients) ● Assessment of tumour heterogeneity and dynamics ● Possibility to perform continuous follow-up examinations	**CLINICAL APPLICATIONS** ● Screening for cancers in high-risk populations ● Early diagnosis of cancer and monitoring early therapeutic response ● Assessment of tumour heterogeneity to guide personalized treatment decisions ● Prediction of metastasis and drug resistance ● Detection of minimal residual disease after surgery/recurrence ● Detection of new cancer driver mutations	**LIMITATIONS** ● Molecular protocols need to be standardized: still too few laboratory applications ● Management of small amounts and easily degradable materials after harvesting, thus requiring extremely sensitive and specific analytic methods ● Poor information on adequate controls ● False-positive and false-negative results: risk of not correctly evaluating the efficacy of a pharmacological treatment. ● Microenvironmental changes may influence the release or the amount of biological materials

Complex data obtained by liquid biopsy should be then integrated in a dedicated database with information derived from tissue biopsy, and ultimately integrated by both pathologists and oncologists to design personalized cancer treatments.

## SERUM MICRORNAS

MiRNAs are short non-coding RNA molecules (22–25 nucleotides in length) able to regulate gene expression, affecting the stability and/or translation of target mRNAs [36, 37]. miRNA biogenesis occurs into the nucleus, where an initial transcript, termed pri-miRNA, is transcribed by a RNA polymerase II; pri-miRNA contains an imperfectly double-stranded region within a hairpin loop and is subsequently cleaved by Drosha nuclease, into a 70–100 bp pre-miRNA. Pre-miRNA is bound by exportin-5 in a complex with Ran and GTP and translocates through the nuclear pore into the cytoplasm, wherein it is cleaved by Dicer in an imperfect double-stranded miRNA of about 20–22 bp. Thereafter, the miRNA duplex is unwound and the mature miRNA strand binds to an Argonaute protein (AGO1-4) into a ribonucleoprotein (RNP) complex, commonly known as RISC, which drives the mature miRNA strand to the 3′-UTR mRNA target sequence. Depending on the degree of complementarity between the miRNA and its target mRNA, miRNA binding to 3′UTR represses translation or induces deadenylation and mRNA decay [38–40]. By regulating the expression of target genes, miRNAs control many biological processes such as cell proliferation, differentiation and apoptosis. An increasing body of data showed how aberrantly expressed miRNAs contribute to the pathogenesis of several types of tumours, acting as oncogenes or tumour suppressors [41].

Several research groups investigated circulating miRNAs for their diagnostic and prognostic potential, suggesting them as excellent blood cancer biomarkers [42]. Unlike intracellular RNA, circulating miRNAs are particularly stable as they are packaged in exosomes or associated with RNA binding proteins. Moreover, miRNAs can be found in body fluids loaded into high-density lipoprotein (HDL), or bound by AGO2 protein outside the vesicles; all of these associations prevent miRNA from being processed by ribonucleases. MiRNAs are also resistant to boiling, pH changes and repeated freeze-thawing cycles which make them very easy to handle. However, although circulating miRNAs are remarkably stable in serum, appropriate and reliable methodologies are recommended during collection and analysis of circulating miRNAs, in order to avoid variability in miRNA assessment [43, 44] .

Several *in vitro* and *in vivo* studies have investigated the role of miRNAs also in OS, underlying their involvement in the onset, progression, and response to chemotherapeutic agents. MiRNAs have gained increasing attention in the management of OS also for their potential applications in diagnosis, prognosis and treatment of this malignancy [44]. Notably, the development of a sarcoma miRNA expression database (S-MED), including OS, identified miRNAs specifically overexpressed or downregulated in specific sarcoma types. MiRNA expression signatures might provide valuable information on sarcoma biology, development of miRNA-based biomarkers, and may improve treatment planning [45]. In particular, several clinical studies investigated the clinicopathological, diagnostic and prognostic value of circulating miRNAs in OS patients’ serum; the results obtained underscored the potential of circulating miRNAs as useful noninvasive biomarkers for early detection and monitoring of OS (Tables 4 and 5, Supplementary Table 1).

**Table 4:** Clinical significance of circulating tumour suppressor miRNA(s) in OS

Tumour suppressor miRNA(s)	Patient cohort	Sample	Clinical observation	Study
miR-326	60 OS pts; 20 HCs	Serum (60 OS & 20 HC) Tissues (30 tumour tissue & 30 adjacent normal tissue)	Lower serum miR-326 levels were found in OS pts compared to HC (0.43-fold decrease, *p* < 0.05). Lower tissue miR-326 levels were found in tumour tissue in comparison to adjacent normal one (≈0.44-fold decrease, *p* < 0.05). Low serum miR-326 levels were associated in OS pts with advanced clinical stage (*p* < 0.05) and distant metastasis (*p* < 0.05), while low tissue miR-326 levels with distant metastasis (p < 0.05). MiR-326 differentiated OS from HC: AUC = 0.897, cut-off value = 1.76, sensitivity = 0.84; specificity = 0.95. Serum miR-326 expression was found to be an independent prognostic factor of unfavorable survival (Cox regression) in OS pts for S: HR = 3.90 [95%CI = 1.13–12. 35], *p* = 0.001.	[49]
miR-133b miR-206	100 OS pts; 100 HC	Serum (100 OS & 100 HC) Tissues (100 tumour tissue & 100 adjacent normal tissue)	Lower serum miR-133b and miR-206 levels were found in OS pts compared to HC (miR-133b: ≈0.42-fold decrease, *p* < 0.001; and miR-206: ≈ 0.50-fold decrease, *p* < 0.001. Lower tissue: miR-133b and miR-206 levels were found in tumour tissue in comparison to adjacent normal one (miR-133b: ≈ 0.58-fold decrease, *p* < 0.001; and miR-206: ≈0.50-fold decrease, *p* < 0.001). MiRNAs levels positively correlated in OS tissues and sera (miR-133b: *r* = 0.56, *p* = 0.01; miR-206: *r* = 0.69, *p* = 0.001). Downregulation of both miR-133b and miR-206 was associated with high tumor grade (p = 0.006), metastasis (*p* < 0.001), recurrence (*p* < 0.001), and the poor response to chemotherapy (p = 0.01). Both serum miR-133b and miR-206 expressions were found to be independent prognostic factors of unfavorable survival (Cox regression) in OS pts for: *S* (miR-133b: HR 5.36 [95% CI = 1.26-11.03], p = 0.02; mir-206: HR 5.42 [95% CI = 1.31-11.28] p = 0.02); and miR-133b/miR-206: HR 9.28 [95% CI = 2.69-20.79] *p* = 0.001), and PFS (miR-133b: HR 5.69 [95% CI = 1.33-11.26], *p* = 0.02; miR-206: HR 5.88 [95% CI = 1.56-12.08], *p* = 0.02; and miR-133b/miR-206: HR 9.69 [95% CI = 2.80-21.82], *p* = 0.001).	[52]
miR-152	80 OS pts; 20 HC; 20 periostitis pts	Serum (80 OS, 20 HC & 20 periostitis) Tissues ((80 OS, 20 HC & 20 periostitis)	Lower serum miR-152 levels were found in OS pts compared to HC and periostitis pts. (≈0.27-fold decrease, *p* < 0.01). Lower tissue miR-152 levels were found in tumour tissue in comparison to adjacent normal or periostitis ones (≈ 0.20-fold decrease, *p* < 0.01); MiR-152 expression was associated with Enneking (*p* < 0.0005) and metastasis (*p* < 0.0005). MiR-152 differentiated OS from HC: AUC = 0.956, cut-off value = 3.5, sensitivity = 0.93; specificity = 0.96. Serum miR-152 expression was found to be an independent prognostic factor of unfavorable survival (Cox regression) in OS pts for S: HR = 0.13 [95% CI = 0.02–0.70], *p* = 0.004.	[53]
miR-95-3p	133 OS pts; 133 HC	Serum (133 OS & 133 HC) Tissues (133 tumour tissue)	Low serum miR-95-3p were found in OS pts compared to HC (0.77-fold decrease, *p* < 0.0001). Low serum miR-95-3p levels were associated with clinical stage (*p* < 0.0005), metastasis (*p* < 0.0005) and response to chemotherapy (*p* < 0.0005). MiR-95-3p differentiated OS from HC: AUC = 0.863. Serum miR-95-3p expression was found to be an independent prognostic factor of unfavorable survival (Cox regression) in OS pts for S: HR = 4.22 [95% CI = 2.31–8.07], *p* = 0.014).	[54]
miR-34b	133 OS pts; 133 HC	Plasma (133 OS & 133 HC) Tissues (133 tumour tissue & 133 adjacent normal tissue)	Lower plasma miR-34b levels were found in OS pts compared to HC (≈0.80-fold decrease, *p* = 0.001). Lower tissue miR-34b levels were found in tumour tissue in comparison to adjacent normal one (≈ 0.20 fold decrease, *p* < 0.0005). Plasma miR-34b levels positively correlated in OS tissues (*r* = 0.21, *p* = 0.004). Lower miR-34b levels were found in metastatic pts compared to non-metastatic ones (*p* = 0.004).	[55]
miR-195	166 OS pts; 60 HC	Serum (166 OS & 60 HC)	Lower miR-195 levels were found in OS pts compared to HC (0.46-fold decrease, *p* < 0.001). Low miR-195 levels were associated with advanced clinical stage (*p* = 0.002), positive distant metastasis (*p* = 0.008). MiR-195 differentiated OS from HC: AUC = 0.892, cut-off value = 1.96, sensitivity = 0.88; specificity = 0.83. MiR-195 expression was found to be an independent prognostic factor of unfavorable survival (Cox regression) in OS pts for S: HR = 5.16 [95% CI = 1.92–11.88], *p* = 0.002), and DFS (HR = 3.62 [95% CI = 1.82–9.09], *p* = 0.01).	[56]
miR-223	112 OS pts; 50 HC	Serum (112 OS & 50 HC)	Lower miR-223 levels were found in OS pts compared to HC (0.31-fold decrease, *p* < 0.01). Lower miR-223 levels were associated with more advanced clinical stage (*p* < 0.001) and positive distant metastasis (*p* < 0.001). MiR-223 differentiated OS from HC: AUC = 0.926, cut-off value = 1.76, sensitivity = 0.90; specificity = 0.97. MiR-223 expression was found to be an independent prognostic factor of unfavorable survival (Cox regression) in OS pts for S: (HR = 4.59, [95% CI = 1.84–11.45], *p* = 0.001).	[57]
miR-497	185 OS pts; 130 HC	Serum (185 OS & 130 HC)	Lower miR-497 levels were found in OS pts compared to HC (≈ 0.27-fold decrease, *p* < 0.001). Lower miR-497 levels were associated with clinical stage (*p* = 0.001), distant metastasis (p = 0.001) and response to chemotherapy (*p* = 0.007). MiR-497 differentiated OS from HC: AUC = 0.848 [95% CI = 0.773–0.923], cut-off value = 0.99. MiR-497 expression was found to be an independent prognostic factor of unfavorable survival (Cox regression) in OS pts for S: (HR = 3.79, [95% CI = 1.99–8.57], *p* = 0.004).	[58]
miR-497	36 OS pts; 26 HC	Serum (36 OS & 26 HC) Tissues (15 stage III tumour tissue & 15 adjacent normal tissue)	Lower miR-497 levels were found in OS pts compared to HC (≈ 0.50-fold decrease, *p* < 0.01). Lower miR-497 levels were found in tumour tissue in comparison to adjacent normal one (≈ 0.60 fold decrease *p* < 0.05).	[171]
miR-125b	138 OS pts	Serum (82 resectable and 56 unresectable)	Lower miR-125b levels were found in unresectable OS pts compared to resectable ones (≈ 0.52-fold decrease, *p* < 0.01). Low miR-125b expression was found to be associated with advanced tumour stages (*p* = 0.006). MiR-125b differentiated chemotherapy-resistant OS from chemotherapy-sensitive OS: AUC = 0.793 [95%CI = 0.664–0.890], cut-off value = 0.61, sensitivity = 0.77; specificity = 0.79. Low miR-125b levels were associated with shorter OS (*p* = 0.049) in unserectable pts and shorter DFS (*p* < 0.001) in resectable ones.	[61]

When no reported in the study, fold increase and decrease in miRNA expression levels between OS pts and HC or between tumour tissue and adjacent normal tissue were calculated as a ratio of reported 2^-∆∆Ct^ (i.e. serum miR-326 2^-∆∆Ct^ in OS / serum miR-326 2-DDCt in HC). Abbreviations: AUC, area under receiving operating characteristics curve; DFS, disease free survival; HC, healthy control/volunteers; HR, hazard ratio; KM, Kaplan-Meier survival curve, L-R, log-rank test; OS, osteosarcoma; PFS, progression free survival; pts, patients; S, overall survival; 95% CI: 95% confidence interval.

**Table 5:** Clinical significance of circulating lncRNAs in OS

Long non coding RNA(s)	Patient cohort	Sample	Clinical observation	Ref.
TUG1	76 OS pts; 36 pts with benign bone tumour; 40 HC	Plasma (29 OS 1 h before and 7 days after surgery; 45 OS after systemic treatment; 18 OS experienced disease progression or relapse; 42 OS newly diagnosed; 36 benign tumour; 40 HC) Tissue (76 tumour tissue & 76 adjacent normal tissue)	Higher plasma TUG1 levels were found in OS pts compared to HC (2.73-fold increase, *p* < 0.01); Higher tissue TUG1 levels were found in tumour tissue in comparison to adjacent normal one (3.46-fold increase, *p* < 0.01). Higher TUG1 levels in pts with disease progression or relapse compared with post-treatment pts (2.71-fold increase, *p* < 0.01). High expression of TUG1 is an independent prognostic factor of unfavorable survival (Cox regression) in OS pts with poor S (HR = 2.78 [95% CI = 1.29–6.00], *p* = 0.009) and PFS (HR = 1.81 [95% CI = 1.01–3.54], *p* = 0.037).	[108]
91H	67 OS pts; 100 HC	Plasma (67 OS & 100 HC)	Higher plasma 91H levels were found in OS pts compared to HC (≈5.0 fold increase, *p* < 0.01). High plasma 91H levels were associated in OS pts with advanced clinical stage (*p* = 0.015), chemotherapy after surgery (*p* = 0.023) and tumour size ≥ 5 cm (*p* < 0.001). High plasma expression of 91H was found to be an independent prognostic factor of unfavorable survival (Cox regression) in OS pts with poor S: higher 91H (HR = 3.14 [95% CI = 1.32–7.49], *p* = 0.010).	[109]
UCA1	151 OS pts; 74 HC	Serum (85 OS &74 HC) Tissues (151 tumour tissue & 151 adjacent normal tissue)	Higher serum UCA1 levels were found in OS pts compared to HC (≈4.80-fold increase, *p* < 0.0001). Higher tissue UCA1 levels were found in tumour tissue in comparison to adjacent normal one (≈6.20-fold increase, *p* < 0.01). High UCA1 levels were associated in OS pts with advanced clinical stage (*p* = 0.001) and metastasis (*p* = 0.007). UCA1 differentiated OS from HC: AUC = 0.831 [95% CI = 0.746–0.916]. High expression of UCA1 was found to be an independent prognostic factor of unfavorable survival (Cox regression) in OS with poor S (HR = 2.52 [95% CI = 1.35–4.83], *p* = 0.011) and PFS (HR = 3.14 [95% CI = 1.66–6.16], *p* = 0.003).;	[112]
ATB	60 OS pts; 60 HC	Serum (60 OS &60 HC) Tissues (60 tumour tissue & 60 adjacent normal tissue)	Higher serum ATB levels were found in OS pts compared to HC (≈10-fold increase, *p* < 0.0001). Higher tissue ATB levels were found in tumour tissue in comparison to adjacent normal one (≈10-fold increase, *p* < 0.0001). High ATB levels was associated in OS pts with advanced clinical stage (*p* = 0.017), metastasis (*p* = 0.037), and recurrence (*p* = 0.020). ATB differentiated OS from HC: AUC = 0.924 [95% CI = 0.876-0.972], sensitivity = 0.83; specificity = 0.90. High ATB levels were associated (KM compared with L-R) with S (*p* = 0.0230) and PFS (*p* = 0.0142).	[113]
MALAT-1	68 OS pts. 40 HC	Serum (46 OS & 46 HC) Tissues (68 tumour tissue & 68 adjacent normal tissue)	Higher serum MALAT-1 levels were found in OS pts compared to HC (≈1.16-fold increase, *p* < 0.001). Higher tissue MALAT-1 levels were found in tumour tissue in comparison to adjacent normal one (≈1.32-fold increase, *p* < 0.001). Higher MALAT-1 levels were associated with tumour size (*p* = 0.008) and distant metastasis (*p* < 0.0005). MALAT1 differentiated OS from HC: AUC = 0.834 [95% CI = 0.738–0.906], cut-off value = 3.68, sensitivity = 0.80; specificity = 0.73. High expression of MALAT1 was associated (KM compared with L-R) with poor S (*p* = 0.02) and PFS (*p* = 0.009).	[116]

When no reported in the study, fold increase and decrease in lncRNA expression levels between OS pts and HC or between tumour tissue and adjacent normal tissue were calculated as a ratio of reported 2^-∆∆Ct^ (i.e. serum 91H 2^-∆∆Ct^ in OS / serum 91H 2^-∆∆Ct^ in HC). Abbreviations: AUC, area under receiving operating characteristics curve; DFS, disease free survival; HC, healthy control/volunteers; HR, hazard ratio; KM, Kaplan-Meier survival curve, L-R, log-rank test; OS, osteosarcoma; PFS, progression free survival; pts, patients; S, overall survival; 95% CI: 95% confidence interval.

In the vast majority of published studies, the main methods used for detecting circulating miRNAs are represented by quantitative real time-PCR (qRT-PCR), gene arrays and sequencing [46].

### Tumour suppressor miRNAs

Table 4 reports the clinical significance of tumour suppressor miRNAs in OS. Deregulation of miR-326 was associated with several physiological and pathological processes; in cancer, miR-326 was associated with bone metastatic progression and chemotherapy resistance by modulating the expression of multidrug resistance-associated protein 1 MRP1 [47, 48]. MiR-326 resulted down-regulated in both OS patients’ serum and tissues, compared with healthy controls and adiacent normal tissues. In particular, miR-326 expression status in serum could strongly discriminate OS from healthy volunteers. The patients with a lower expression of miR-326 tended to have distant metastasis and advanced tumour stage, as well as a shorter overall survival compared to patients with high miR-326 serum expression. In view of these results, the authors indicated that miR-326 might represent an independent prognostic biomarker for overall survival in OS, particularly for patients with advanced disease. Furthermore, by *in vitro* assays, the authors investigated the role of miR-326 in OS invasion and apoptosis, which was dependent on the targeting of the tumour apoptosis inhibiting Bcl-2 gene [49].

MiR-133b and miR-206, two muscle-specific miRNAs, were found to be down-regulated in OS cell lines, where they negatively interfered with cell proliferation and invasion, while promoting apoptosis; conversely, miRNA overexpression reverted these phenotypes [50, 51]. Serum and tissue levels of both miR-133b and miR-206 in OS patients were positively correlated and markedly lower than those, respectively, in healthy volunteers and non cancerous bone tissues. Thereafter, the potential prognostic value of the circulating miRNAs was evaluated in serum: low expression levels of both miR-133b and miR-206 were closely related to a shorter overall and disease-free survival; conversely, patients with high miR-133b/miR-206 ratio had a better prognosis. Low miR-133b and low miR-133b/miR-206 were also associated with poor response to chemotherapy. Thus, miR-133b, miR-206 independent levels, or in combination, were all independent prognostic factors of a worse survival in OS [52].

Similarly, down-regulation of several serum miRNAs (miR-152, miR-95–3p, miR-34b, miR-195, miR-223, miR-497) was associated with OS progression and poor prognosis, suggesting their value as potential serum biomarkers for early detection and clinical evaluation in patients with OS [53–58].

Despite the rapid development and use of multi-agent chemotherapy regimens, chemoresistance remains the major cause of treatment failure in the management of OS; thus, an early diagnosis of OS, as well as the identification of effective biomarkers predictive of chemoresistance remain crucial for timely and appropriate treatments.

Several studies showed that miRNA deregulation was closely associated with the development of chemoresistance, also in OS [59]. Notably, miR-125b overexpression improved sensitivity of OS cells to cisplatin treatment by targeting the anti-apoptotic protein Bcl-2 [60]. In the study by Luo Z et al., the correlation between serum miR-125b levels and response to therapy was evaluated in OS patients, which were previously divided in two groups: resectable (received cisplatin-based neoadjuvant and adjuvant chemotherapy and surgery) and unresectable (received cisplatin-based aggressive chemotherapy). In addition, the authors defined chemotherapy sensitivity as complete or in partial remission, while disease progression as sign of chemotherapy resistance. For the resectable group, OS patients with low circulating miR-125b levels experienced shorter disease-free survival, parameter used by the authors to indirectly assess patients’ response to chemotherapy. Further, a negative correlation between miR-125b expression and tumour response to cisplatin-based therapy was reported for the unresectable group, where low serum miR-125b levels were associated with advanced tumour stages. Overall, the authors argued that down-regulation of circulating miR-125b might predict poor prognosis and response to cisplatin-based chemotherapy [61].

### OncomiRNAs

Up-regulation of oncogenic miRNAs in OS was associated with the development and progression of this malignancy, suggesting them as attractive potential target for OS therapy. Several studies found a consistent group of circulating miRNAs markedly higher in OS patients than healthy control; moreover, data regarding the association between miRNA levels and clinicopathological features of OS patients showed that the majority of up-regulated miRNAs positively correlated with advanced clinical stage, large tumour size, distant metastasis and, in some cases, chemotherapy resistance [62, 63]. Supplementary Table 1 reports the clinical significance of circulating oncomiRNAs in OS.

Ouyang et al., reported the prognostic utility of the combination of different circulating miRNAs rather than a single miRNA. The expression levels of six candidate miRNAs aberrantly expressed in OS tissues and cell lines, were further evaluated by qRT-PCR in plasma of patients, opening the possibility of using them as non-invasive biomarkers of disease; three miRNAs were considered relevant and validated in 40 OS patients and 40 healthy controls. Consistently, detection of a three-plasma miRNA signature (miR-21, miR-143 and miR-199a-3p) in OS allowed to discriminate diseased from healthy patients with higher accuracy than using individual miRNA. In particular, circulating levels of miR-21 resulted higher in OS patients than controls, while miR-199a-3p and miR-143 were decreased. Evaluation of tumour metastatic status and histopatological subtype highlighted higher level of miR-21 in metastatic compared with non-metastatic patients, while miR-143 had the opposite trend. Similarly, miR-21 levels were significantly increased in osteoblastic compared with non-osteoblastic patients [64].

Likewise, high miR-196a and miR-196b levels, as well as the combined upregulation of circulating miR-196a/miR-196b in OS patients were all considered independent prognostic factor for overall survival and disease free survival. Significant prognostic differences were found among four different groups in relation to miR-196a/miR-196b co-expression, with miR196a-high/miR196b-high OS patients having the worst prognosis (high tumour grade, presence of metastasis and recurrence of OS patients). Additionally, the levels of those two miRNAs in OS tissues positively correlated with those in patients’sera [65].

TaqMan low-density qPCR (TLDA) technique was used to analyze expression levels of 739 miRNAs in pooled samples from pre- and post-surgery OS patients, and from healthy controls. The results obtained showed that miRNA levels varied between pre-surgery and healthy controls, and also between pre- and post-surgery. Moreover, selected miRNAs were validated by individual qRT-PCR generating a list of 4 miRNAs, including miR-195–5p, miR-199a-3p, miR-320a and miR-374a-5p, whose combined co-expression discriminated, with high sensitivity and specificity, OS patients from healthy volunteers. Subsequently, the levels of the 4 miRNAs resulted significantly reduced in the plasma after surgical resection, while, correlation analysis between circulating miRNAs and clinical factors, pointed out that miR-195–5p and miR-199a-3p correlated with metastatic status, while miR-320a and miR-374-5p correlated with histological subtype [66].

A similar experimental approach led to identify miR-199a-5p as blood biomarker for pre- and post-operative OS patients. In detail, the authors found significantly high levels of miR-199a-5p in pre-operative OS patients compared with healthy controls; on the other hand, miRNA levels decreased in post-operative compared with pre-operative samples, reaching levels comparable with healthy controls. The proposed study indicates miR-199a-5p as a powerful biomarker useful for monitoring disease recurrence during post surgery follow-up. Differences in the circulating miRNA levels, secreted by OS cells, strongly correlated with pre and post-operative status, when surgery resection of the tumour seems to induce a drastic reduction of miR-199a-5p released in the bloodstream [67].

Up-regulation of miR-300 was found in OS tissues and cell lines compared with paired adjacent non-cancerous bone tissues and osteoblastic cells. MiR-300 is known to promote cell proliferation, invasion and epithelial-mesenchymal transition in OS cell lines [68]; in an attempt to assess the diagnostic value of circulating miR-300, its expression was studied and found strongly up-regulated in OS patients and positively correlated with expression levels of its tissue counterpart. Similarly to miR-199a-5p, serum miR-300 levels decreased in OS patients following curative surgery. High levels of miR-300 were detected in advanced clinical stages and in metastasis, and indicated as an independent predictor factor for poor overall survival and progression free-survival rate in OS [69]. Of note, the authors underscored an important limitation of using miR-300 as a single biomarker for early detection and monitoring of OS: in fact, circulating miR-300 has been involved in the pathogenesis of other cancers, such as glioma and head and neck squamous cell carcinoma [70, 71].

The miRNA-29 family is composed by three members: miR-29a, miR-29b and miR-29c; there is a large body of literature documenting the aberrant expression of these miRNAs in various cancers, with both tumour suppressors and oncogenic roles in a cellular context-dependent fashion [72, 73]. In a physiological setting, miR-29 was found to control osteoblast differentiation by targeting osteonectin, a key factor in bone remodelling; furthermore, miR-29 was reported to play a role in muscle cells development [74, 75]. *In vitro* models of multiple myeloma (MM)-related bone disease showed that miR-29b enforced expression counteracted osteoclast differentiation promoted by cancer cells, providing a rationale for a potential novel targeted therapy for MM-related bone disease [76]. Several studies reported deregulation of miR-29 family members in human OS cell lines, although data from different studies are somehow controversial [77, 78]. In OS patients, high expression levels of miR-29a, miR-29b and miR-29c were detected in tumour tissues and patients’sera, compared with the corresponding adjacent normal tissues or with healthy controls, respectively. In addition, serum and tissue levels of the three miRNAs positively correlated. However, only high miR-29a and miR-29b expression levels correlated with tumour grade, positive metastasis and disease recurrence, as well as with shorter overall and disease-free survival [79].

Global miRNA expression analysis identified 236 miRNAs as highly expressed in OS patients compared with healthy controls. Among these, eight miRNAs were detected in the culture medium of OS cell lines, meanwhile, qRT-PCR analysis confirmed upregulation of miR-25-3p and miR-17-5p in both OS cells and their derived exosomes. The authors, then, focused on circulating miR-25-3p showing its capability to strongly discriminate OS from non-OS patients (other sarcomas), and from healthy volunteers. Elevated expression of circulating miR-25-3p correlated with clinical distant metastasis and was useful for tumour monitoring during multi-modal treatment, although few cases were analyzed. Likewise, in tumour-bearing mice, miR-25-3p serum levels increased with tumour growth, decreased after tumour resection and, in mice with lung metastasis despite resection, miRNA level increase was revealed again [80].

In OS tissues and cell lines, miR-17 acts as oncomiR by targeting phosphatase and tensine homolog (PTEN), a relevant tumour suppressor gene in cancer. PTEN inactivation or loss of function is found in several tumours, where consequently phosphoinositide 3-kinase (PI3K)/AKT signaling pathway results activated thus promoting cancer progression. In OS cell lines, silencing of miR-17 leads to increased PTEN expression with decreased cell proliferation, migration and invasion [81]. High PTEN tissue expression frequently occurred in OS patients with a better prognosis, while upregulation of circulating miR-17 was associated with a poor prognosis. Of note, the authors observed a positive correlation among miR-17 serum and tissue levels in OS patients, while circulating miR-17 levels inversely correlated with PTEN tissue expression; notably, the negative correlation between miR-17 and PTEN expression was significantly associated with poor survival in OS patients [82].

Altered levels of miR-221 have been observed in several tumours [83, 84]; in human OS cell lines, over-expression of miR-221 induced cell survival and cisplatin resistance at least partly through targeting the PI3K/PTEN/Akt pathway [85]. More interestingly, the diagnostic value of miR-221 was then evaluated in OS patients. The levels of miR-221 were elevated in OS serum and tissue, compared to healthy counterparts; moreover, circulating miR-221 could efficiently discriminate OS patients from healthy volunteers. High circulating miR-221 levels were positively associated with clinical stage and distant metastasis; moreover, miR-221 level was described as an independent prognostic factor for overall survival in OS patients [86].

The onco-miR-27a is abnormally expressed in multiple cancers promoting tumour growth and metastasis [87-89]. Previous studies evaluated the impact of miR-27a overexpression on the metastatic potential of OS cell lines; in detail, enforced expression of miR-27a sustained cancer cell migration and invasion. Furthermore, miR-27 acts as an oncogene by targeting the tumour suppressor gene mitogen activated protein kinase 4 (MAP2K4); in turn, MAP2K4 regulates JNK or p38 phosphorylation promoting OS cancer progression [90]. Notably, Tang J et al., observed high miR-27a serum levels in OS patients compared to healthy controls, confirming its strong correlation with aggressive disease (advanced clinical stage and positive distant metastasis) and poor response to chemotherapy. Moreover, circulating miR-27a expression in OS patients was an independent and significant prognostic factor to predict overall survival and disease-free survival [91].

The identification of novel miRNA-based regulatory pathways involved in apoptosis and chemotherapy resistance may provide new promising approaches in OS management. Recently, upregulation of miR-24 levels in OS patients’ serum and tissues, when compared with healthy controls and non-cancerous tissues, was shown to be closely related to drug resistance. Based on *in vitro* studies, such research highlighted novel mechanisms underlying resistance to doxorubicin (DOX), the most common drug used to treat OS. In detail, inhibition of miR-24 could overcome chemoresistance, increasing sensitivity of DOX-resistant OS cell lines to chemotherapy. MiR-24 silencing led to upregulation of the pro-apoptotic protein BIM, a member of the Bcl-2 family, and promoted the release of apoptotic factors (such as Smac/DIABLO) from mitochondria, with consequent activation of caspases-dependent cell death. Data obtained showed the miR-24-BIM-Smac-DIABLO axis as a novel therapeutic target to enhance OS cell response to DOX, with potentially important consequences on OS management [92].

Despite the crucial role of miRNAs in various biological processes (including proliferation, differentiation, apoptosis) and their documented dysregulation in human cancer, the real clinical uselfulness of miRNAs is under debate.

As previously reported, the recent literature indicates circulating miRNAs as tumour-associated biomarkers useful for early cancer detection, and also for assessing tumour dynamics and drug sensitivities. However, some critical issues in using circulating miRNAs as disease biomarkers need to be considered. In fact, even if miRNAs are considerably stable in clinical plasma samples, it is mandatory to develop standardized protocols for sample collection (from whole blood collection to plasma/serum preparation), miRNA extraction, quantification and data analysis, since pre-analytical and analytical steps can substantially influence miRNA recovery [93].

Additionally, it appears that individual variability, such as race and gender, as well as environmental factors and life-style, such as drug assumption, smoking and nutrition could affect overall circulating miRNA profiles [94–99]. All these variables may finally alter the profile of the single circulating miRNA analyzed, leading to irreproducible results and/or difficult interpretations. Therefore, a more rigorous approach in the study of disease-related biomarkers is recommended, based on extremely sensitive and specific analytic methods as well as on the monitoring of multiple miRNAs in plasma of patients, in combination with common clinicopathological parameters.

## SERUM LONG NON-CODING RNAS

LncRNAs are non-protein coding transcripts longer than 200 nucleotides, implicated in a plethora of biological functions, such as development, differentiation, as well as in regulating gene expression [100]. In cancer, lncRNAs have been widely described for their ability to interfere with oncogenic and tumour-suppressing pathways; among circulating nucleic acids in the blood plasma of cancer patients, the clinical significance of lncRNA levels is increasingly emerging. Notably, several studies detected lncRNAs in body fluids as, for example: PCA3 in prostate cancer, H19 in gastric cancer, HULC in hepatocellular carcinoma, MALAT-1 in non-small cell lung cancer [101–104].

Similar to circulating miRNAs, plasma-derived lncRNAs are found in a stable form protected from endogenous RNases or encapsulated within microvesicles, such as exosomes. Nevertheless, accurate techniques to collect, storage and process samples need to be standardized and commonly accepted. In addition, researchers are evaluating the influence of individual and environmental factors on lncRNAs expression levels, in order to establish them as real disease biomarkers. To be sure that changes in expression levels of circulating lncRNA can be used to monitor cancer patient, it will be necessary to develop specific circulating lncRNA signatures unique to individual cancer types compared with healthy subjects [105].

LncRNAs were reported to be involved also in OS pathogenesis, metastasis and chemoresistance. A microarray analysis explored the expression profile of lncRNAs in human primary OS and their noncancerous counterparts; the study highlighted more than 25.000 lncRNAs deregulated, with both oncogenic and tumour suppressive transcripts. Notably, lncRNAs were functional correlated with several signaling pathways implicated in OS proliferation and metastasis [106, 107]. Table 5 reports the clinical significance of circulating lncRNAs in OS.

Taurine up-regulated gene 1 (TUG1) is a potential oncogenic lncRNA that has been shown to support OS cell proliferation and inhibit cell apoptosis. High TUG1 levels were detected in OS tissues when compared with corresponding adjacent normal tissues; in addition, OS patients with high tissue TUG1 levels had a significantly worse prognosis, and TUG1 resulted as an independent prognostic marker for both overall and progression-free survival. The serum levels of TUG1 increased in pre-operative patients compared to healthy controls, while it decreased following curative surgery; in particular, its expression levels increased again in patients experiencing disease progression or relapse [108].

Circulating expression profile of 91H, a lncRNA located on the position of the H19/insulin-like growth factor 2(IGF2) locus, was investigated in the serum of OS patients and in healthy controls; interestingly, 91H overexpression was correlated with advanced clinical stage, chemotherapy after surgery and tumour size (when greater than 5 cm). Finally, 91H overexpression predicted poor overall survival, and was indicated as an independent prognostic factor [109].

LncRNA UCA1 (urothelial carcinoma associated 1) was originally investigated in human bladder cancer; later, its deregulated expression was demonstrated in several other tumours [110, 111]. High expression levels of UCA1 were found in OS tissues and patients’ sera when compared with controls; in addition, expression of circulating UCA1 significantly correlated with clinical stage and metastasis. Importantly, the authors demonstrated that UCA1 was an independent prognostic factor predicting overall survival and disease free survival in OS patients [112].

LncRNA activated by transforming growth factor-β (lncRNA-ATB) has been described in several cancers, including recently OS [113]. Compared with normal controls, the expression levels of circulating lncRNA-ATB in OS patients resulted significantly increased, and were able to accurately discriminate OS patients from healthy controls. Upregulation of circulating lncRNA-ATB was found associated with poor prognosis, while its tissue expression correlated with advanced Enneking stage and metastasis. To investigate the biological role of lncRNA-ATB, *in vitro* assays using ectopically expressed lncRNA-ATB revealed an increase in OS cell proliferation and migration ability; conversely, lncRNA-ATB silencing induced opposite effects. Notably, overexpression of lncRNA-ATB decreased miR-200s while up-regulated its two major targets, zinc finger E-box binding homeobox proteins ZEB1 and ZEB2, finally affecting OS progression [113].

The metastasis associated lung adenocarcinoma transcript 1 (MALAT1), was firstly described as a predictive biomarker for metastasis in the early stage of non-small cell lung cancer and then in other cancers [104]. In OS, MALAT1 promotes proliferation and metastasis of cancer cells by activating the PI3K/Akt pathway [114]; additionally, elevated tissue levels of MALAT1 correlated with advanced clinical stage and distant metastasis, contributing to a shorter survival time [115]. More recently, it was demonstrated that MALAT1 is activated by the transcription factor TGF-β and, then, promoted OS metastasis through EZH2-induced suppression of E-cadherin. High MALAT1 serum levels were then associated with tumour size and distant metastasis, but also with poor overall survival and progressive free survival compared with the low expressing patients [116].

## SERUM CIRCULATING TUMOUR CELLS

Development of distant metastases in OS patients originates by spread of cancer cells through the blood vasculature or lymphatic system. Circulating tumour cells (CTCs) are currently considered as a biomarker of disease progression, since they well correlate with the status of metastatic cancer. Appropriate isolation and detection methods allow to monitor the presence and number of CTCs before and after chemotherapy treatment, which likely represent important predictors of prognosis for cancer patients. Furthermore, the analysis of genetic alterations found in CTCs can be used to drive therapeutic decisions. However, CTCs isolation and characterization techniques need to be thoroughly improved in terms of molecular features of the cells, given their fragility, high heterogeneity and the very small amount recovered from the blood [117, 118].

Detection of live CTSc requires highly sensitive and specific methods, usually preceded by an initial step to isolate and enrich tumour cells from blood. Enrichment of CTCs from a large blood volume is a necessary technique, since there is about 1 CTC for 10^6^ mononuclear cells. To date there are two main enrichment techniques which include, respectively, physical (including cell size, or electrical charge) and immunomagnetic, antigen-dependent methods. Filtration methods rely on physical properties that allow the isolation of CTCs by size; in detail, since CTCs are larger in size than hematopoietic cells, only the blood cells pass through the pores (7.5 to 8 μm in diameter) of the filters, whereas approximately 85–100% of CTCs are retained. After isolation, CTCs can be stained (Hematoxylin-eosin or May-Grunwald-Giemsa) and identified using morphologic/cytopathologic criteria. The immunomagnetic antigen-dependent technique selects CTCs by expression of membrane proteins; it is usually performed by positive enrichment of the epithelial cell markers EpCAM and keratins, and also combined with negative depletion of hematopoietic CD45 positive cells [117, 119, 120].

Due to the mesenchymal origin of OS tumours, selection methods based on expression of epithelial-specific markers are not suitable; therefore, CTCs from OS tumours have been commonly selected by cell size. Screening of cell-surface markers differently expressed on cancer and normal cells provides a convenient method for analyzing CTCs. Vimentin is an intermediate filament highly expressed in mesenchymal cells and correlated with sarcoma migration and invasiveness. Vimentin overexpression was frequently observed in several cancers, and a cell-surface vimentin emerged as an exclusive marker for different subtypes of sarcoma, including OS. The proposed technique exploited a novel monoclonal antibody specifically recognizing a tumour-surface vimentin, thus discriminating cancer cells from mononuclear blood cells that express intracellular vimentin [121, 122].

In a pilot study, investigators evaluated the expression of Collagen I (Col1) by semiquantitative RT-PCR in both OS patients and healthy controls, correlating mRNA levels with patients’s clinical outcome. Notably, they reported elevated Col1 transcript levels in peripheral blood samples from OS patients compared with healthy controls. Furthermore, OS patients characterized at diagnosis by elevated Col1 mRNA levels developed clinical metastases within a year; conversely, no metastases were found in OS patients characterized by low Col1 mRNA transcript levels. Although the number of patients was low, the authors highlighted the potential diagnostic and prognostic values of Col1 mRNA, being the molecular approach a minimally invasive method to follow up patients after OS diagnosis [123].

A polymerase chain reaction assay based on an enzyme-linked immunosorbent assay (PCR-ELISA) was developed in order to detect OS CTCs in a mouse metastatic model. The authors used Osf2/Cbfa1, also called Osf2, as target gene. It is a member of the transcription factors *runt* involved in osteoblastic differentiation, whose expression is restricted in cells of osteoblastic lineage. In particular, the expression of a splicing variant of *Osf2* was limited to normal bone and OS cells, meanwhile Osf2 mRNA was found highly expressed in the blood of metastatic mice compared with the blood of healthy mice [124].

Ezrin, an Ezrin/Radixin/Moesin protein family member, is a linker protein connecting the actin cytoskeleton with plasma membrane. Several studies have demonstrated the involvement of ezrin in sarcoma development and metastasis, describing its association with clinicals parameters of disease progression. Recently, ezrin expression was detected by multiple RNA-*in situ* hybridization (RNA-ISH) assay in CTCs isolated by peripheral blood of OS patients; of note, the expression of ezrin was higher in stage IIIB than stage IIB (Ennekin staging system: Table1), supporting the hypothesis that ezrin levels positively correlated with distant metastasis [125–128].

More recently, a prospective clinical study described a new method to quantify OS CTCs from blood; this is based on abnormal chromosome numbers (aneuploidy) in CTCs instead of surface markers. Aneuploidy was analyzed by fluorescence *in situ* hybridization (FISH) using fluorescence-labeled alpha-satellite probes for the centromeres of chromosome (CEP 8). Notably, patients with metastatic OS had more CTCs, and correlative analysis between number of CTCs and progression-free survival revealed a worse prognosis for patients with high CTCs levels [129].

## SERUM CIRCULATING TUMOUR DNA 

Cell-free circulating tumour DNA (ctDNA) can be considered as a potential surrogate for the entire tumour genome, as analysis of ctDNA for somatic mutations may be a way to detect and follow the progression of a patient’s tumour. The mechanism by which cell-free ctDNA is released into the circulation is still unclear, although these fragments may possibly derive from the primary tumour site, as well as from metastases, or even from apoptotic CTCs [119, 130, 131]. ctDNA released into the blood harbours specific genomic alterations detected in primary tumour; additionally, its somatic genetic alterations reflect the spatial and temporal heterogeneity observed between primary and metastatic tumours, overcoming limitations of single tissue biopsy. Following the recent advances in cancer genome project (CGP) and next generation sequencing (NGS) technology, analysis of genetic lesions by ctDNA might become a reality, despite the small amount of this circulating ctDNA in the blood [132, 133]. Thus, ctDNA as liquid biopsy may represent an alternative approach for tumour diagnosis, prognosis and personalized therapy, even in OS patients.

In a recent study on primary breast tumours, not all the mutations identified in the metastasis could be detected in the primary lesion; conversely, all mutations identified in plasma-derived DNA were accurately detected (by sequencing analysis of DNA) in tissue biopsies of both primary breast cancer and its synchronous liver metastasis [134]. Importantly, the authors demonstrated that ctDNA analysis is able to capture the heterogeneity of primary tumour and metastasis. One of the most commonly used liquid biopsy analysis is the search of epidermal growth factor receptor (EGFR) mutations, which predicts responsiveness to EGF receptor (EGFR)-tyrosine kinase inhibitors in a distinct clinicopathologic subset of non-small-cell lung cancer patients [135].

Due to the rarity of OS disease, the research on ctDNA in OS patients is still preliminary, especially if compared with other tumours. Nevertheless, ctDNA analysis could provide useful information, given the high degree of genetic heterogeneity in OS [136].

A pilot study characterized ctDNA from the plasma of SCID mice injected with OS cells; in particular, DNA extracted was subjected to NGS using custom designed probes for cancer-related genes in OS (TP53, RB1, MET and PTEN). The authors detected over 1000 mutations, already identified as OS-specific mutations, and concluded that the study provided the rationale to use ctDNA within clinical trials [Fremed M, Piperdi S, Zhang W, Maqbool S, Calder B, Castellanos R, et al. Abstract 3133A: Circulating tumour DNA as ‘liquid biopsy’in pediatric osteosarcoma. Cancer Research, American Association for Cancer Research-AACR; 2016 Jul 15;76(14 Supplement): 3133A-3133A. Available from: http://dx.doi.org/10.1158/1538-7445.am2016-3133a].

CtDNA may provide information not only for genetic aberrations but also for epigenetic alterations of cell-free DNA. Epigenetic alterations, including changes in DNA methylation and associated histone modifications, are closely related with tumour development and disease progression [137]. CtDNA from cancer patients can account for genetic/epigenetic modifications, because nucleic acid fragments are shed into the circulation from all tumours in a patient’s body; notably, differential methylation status of tumour-associated genes matches among ctDNA and corresponding tumour tissues [138]. In OS cell lines, treatment with decitabine, an inhibitor of DNA methylation, led to the upregulation of several genes some of which, like GADD45A, restored apoptotic response [139]. In a recent study, two syngeneic human OS cell lines, derived respectively from a primary tumour and a skip metastasis in the same patient, were established. By performing a methylated DNA immunoprecipitation (MeDIP) assay in combination with expression profiling, the authors found a higher increase in Iroquois homeobox 1 (IRX1) expression in highly metastatic OS cells compared with nonmetastatic OS cells. They later confirmed elevated IRX1 expression in metastatic OS patients due to hypomethylation of its own promoter. In OS cell lines, the promoter hypomethylation of the *IRX1* gene leads to IRX1 overexpression, which in turn exerts its pro-metastatic effects increasing invasion, anoikis resistance *in vitro* and lung metastasis *in vivo*. Conversely, in *in vitro* assays, downregulation of IRX1 in OS cells resulted in decreased CXCL14 expression levels, inhibition of NF-κB activity and suppression of metastasis. The analysis of the methylation status of *IRX1* promoter in the serum of primary OS patients highlighted that hypomethylated *IRX1* correlated with worse lung metastasis-free survival, thus suggesting *IRX1* hypomethylation as a potential biomarker for early detection of lung metastasis in OS patients [140].

## SERUM EXTRACELLULAR VESICLES

The role of cancer-derived exosomes as novel cell-cell signals during tumour growth and progression has been recently highlighted. Exosomes released by cancer cells may affect survival, apoptosis, invasion, angiogenesis and resistance to chemotherapy, and may also prepare the metastatic niche [141, 142]. These nanovesicles (40–150 nm) of endocytic origin actively transport and horizontally transfer information such as proteins, miRNAs and mRNAs to target cells, thus influencing their behaviour and strongly modifying the entire microenvironment. Several reports demonstrated that exosomes enter into a variety of body fluids including blood, where the amount of tumour-derived exosomes is high and increase during tumour progression. Circulating exosomes are emerging as clinically useful tools for cancer detection and high levels of cancer cell derived-exosomes were found in the plasma of patients with breast, colorectal, ovarian and prostate cancers [143–145]. The bioactive molecules carried by exosomes strongly reflect the dynamic changes of the producing cells, particularly during metastatic progression and pharmacological response [146]. Even if standardization procedures in exosomes’ isolation and characterization still remain a major challenge, it is well accepted that exosome content is heterogeneous and compatible with the pathological state of their cells of origin, thus making exosome promising biomarkers deserving in-depth investigation.

Therefore, in the last decade researchers have tried to develop circulating exosomes-based biomarkers for use in routine clinical practice. The conventional methods of exosomes isolation allow to analyze nanovesicles but also their molecular content, such as proteins, DNA and miRNAs. To date, the commonly used methods include ultracentrifugation, density-gradient separation, size exclusion chromatography, immunoaffinity capture methods as well as exosomes precipitation and microfluidics-based isolation techniques [147, 148]. Moreover, new alternative approaches, relying on the expression of surface tumour markers, are being developed in order to discriminate tumour-derived exosomes from those secreted from healthy cells. Recently, a novel selective capture methodology was reported for exosomes expressing the prostate-specific membrane antigen (PSMA) biomarker, critical for early diagnosis, prognosis and treatment design of prostate cancer [149].

Several studies reported that analysis of tumour-specific antigens by circulating exosomes could improve the sensitivity and specificity to detect and monitor cancer patients. Alegre E et al. detected MIA (Melanoma Inhibitory Activity) and S100B, two markers commonly used to follow patients with advanced melanoma, in circulating exosomes from melanoma patients; their serum quantification could have a diagnostic and prognostic value, as significantly higher levels of both markers have been observed in melanoma patients compared to healthy controls [150]. The membrane-associated protein Glypican-1, described as overexpressed in pancreatic cancer, was found in circulating exosomes of pancreatic cancer patients, distinguishing with good specificity and sensitivity, healthy controls and patients with benign disease from those with early and advanced stages disease. High levels of glypican-1 correlated with metastatic disease burden and the survival of pre- and post-surgical patients [151]. More recently, Liu et al. proposed circulating exosomal miR-23b-3p, miR-10b-5p and miR-21-5p as non-invasive prognostic biomarkers of non-small-cell lung cancer, reporting a poorer overall survival in patients with high expression levels of the three exosomal miRNAs [152].

In OS, similarly to ctDNA, circulating exosomes only now begin to be evaluated within clinical studies enrolling OS patients. Indeed, the specific contents of OS-derived exosomes, their contribution to tumorigenesis as well as their role in drug resistance are beginning to be explored by *in vitro* and *in vivo* studies. For instance, it has been shown that OS cell lines actively secrete exosomes, which contain many proteins related to biological functions and specifically involved in tumour growth and metastasis. Furthermore, gene ontology analysis of exosomal and non-exosomal fractions revealed also differences in the enrichment of functional categories involved in tumorigenesis [153].

OS-derived extracellular vesicles (OS-EVs) contribute to establish a metastatic niche through induction of IL-6 production by mesenchymal stem cells (MSC), which in turn sustain tumour growth and progression. In an attempt to explore the underlying mechanisms, the authors found that an EV-associated form of TGFβ was responsible of IL-6 production in MSC. In addition, by *in vivo* studies, “tumour-educated” MSC (TEMSCs) injected in a pre-clinical mouse model supported OS growth and lung metastasis formation, while co-administration of tocilizumab, an interleukin-6 antibody, strongly reduced the effects induced by TEMSCs. Further analysis revealed that serum levels of EV-associated TGFβ were significantly higher in OS patients compared to healthy controls, supporting a clinical significance [154].

Data obtained *in vitro* demonstrated that doxorubicin-resistant OS cells may transfer their drug- resistance to sensitive cells by releasing exosomes. In particular, exosomes were purified by multidrug resistant human OS MG-63DXR30 (Exo/DXR) and MG-63 parental cells (Exo/S); incubation of OS cells with Exo/DXR decreased the sensitivity of parental cells to doxorubicin, while treatment with Exo/S was irrelevant. Moreover, multidrug-resistance P-glycoprotein mRNA expression increased in MG-63, after incubation with Exo/DXR. Overall, these results indicate that tumour-derived exosomes act as vehicles allowing the exchange of biological cargo, specifically contributing to drug-resistance mechanisms by transferring the intercellular transfer of MDR-1 mRNA and its product P-glycoprotein [155].

Profiling analysis of exosomal miRNA levels from plasma of OS patients revealed a differential response to chemotherapy, with 12 miRNAs upregulated and 18 miRNAs downregulated in exosomes isolated from patients who had poor response to chemotherapeutic treatment when compared to those with a better status. Validation of differential miRNAs using an independent cohort of 20 OS patients with poor response, 20 OS patients with good response and 20 healthy controls confirmed miR-124, miR-133a, miR-199a-3p and miR-385 as significantly reduced in patients who have a poor response to chemotherapy when compared with good responders. Instead, miR-135b, miR-148a, miR-27a and miR-9 resulted highly expressed in circulating exosomes from good responder patients. Data obtained showed that a specific subset of circulating exosomal miRNAs, could be used as reliable diagnostic markers to highly discriminate OS patients with poor chemotherapeutic response from those with good response [156].

## CONCLUSIONS

Osteosarcoma is a rare and highly heterogeneous bone cancer, resulting from the interplay of environment and genes; it occurs most often in young adults and strongly weakens their quality of life. Despite the management of OS has been evolved in the last years, the timely diagnosis and staging of the disease represents a primary prerequisite for successful surgical and pharmacological treatments. Currently, tissue biopsy and imaging are the most common diagnostic tests used by clinicians to detect and monitor treatment for OS; however, obtaining tissue biopsy may be sometimes difficult due to the location or to the small size of the tumour.

The identification of prognostic factors remains a challenge of OS treatment; indeed, the prognostic significance of biomarkers proposed to date is still controversial among the orthopaedic oncologists. In alternative, liquid biopsy represents a non-invasive and time-saving approach that may provide crucial information on early detection, therapeutic decision and response to therapy in OS. Notably, liquid biopsy already represents a reality in clinical practice, as demonstrated by the EGFR genetic testing for non-small cell lung cancer [135].

In spite of several attempts to propose blood-based biomarkers for the clinical management of OS, few biomarkers have already been validated in statistically significant prospective trials. Notably, the studies reported in this manuscript have identified several promising disease biomarkers; data obtained are therefore very encouraging and, if further validated, may provide good candidates usable in non-overlapping biomarker panels for OS. Anyway, the development of such unique and reliable biomarkers must be supported by standardization procedures throughout all phases, from biomarkers discovery to clinical validation. Moreover, it is advisable that the methodologies are validated in multi-center prospective clinical trials with a large cohort of patients. Indeed, a panel of biomarkers should be combined with patients’ clinical data to draw a real-life clinical scenario [157, 158].

## SUPPLEMENTARY MATERIALS TABLE




