# Supplementary material for: Circulating biomarkers in osteosarcoma: new translational tools for diagnosis and treatment
Source: Oncotarget. 2017 Aug 3;8(59):100831–51. doi: 10.18632/oncotarget.19852 (PMC5725068; doi:10.18632/oncotarget.19852)
Supplement: Supplementary file 1 [file oncotarget-08-100831-s001.pdf]

Circulating biomarkers in osteosarcoma: new translational tools for diagnosis and treatment

SUPPLEMENTARY MATERIALS

Supplementary Table 1: Clinical significance of OncomiRNA(s) in OS. See_Supplementary_Table 1

REFERENCES

1. Ouyang L, Liu P, Yang S, Ye S, Xu W, Liu X. A three-plasma miRNA signature serves as novel biomarkers for osteosarcoma. Med Oncol. 2013; 30:340.
2. Zhang C, Yao C, Li H, Wang G, He X. Combined elevation of microRNA-196a and microRNA-196b in sera predicts unfavorable prognosis in patients with osteosarcomas. Int J Mol Sci. 2014; 15:6544–55.
3. Lian F, Cui Y, Zhou C, Gao K, Wu L. Identification of a plasma four-microRNA panel as potential noninvasive biomarker for osteosarcoma. PLoS One. 2015; 10:e0121499.
4. Zhou G, Lu M, Chen J, Li C, Zhang J, Chen J, Shi X, Wu S. Identification of miR-199a-5p in serum as noninvasive biomarkers for detecting and monitoring osteosarcoma. Tumour Biol. 2015; 36:8845–52.
5. Xue Z, Zhao J, Niu L, An G, Guo Y, Ni L. Up-Regulation of MiR-300 Promotes Proliferation and Invasion of Osteosarcoma by Targeting BRD7. PLoS One. 2015; 10:e0127682.
6. Liu JD, Xin Q, Tao CS, Sun PF, Xu P, Wu B, Qu L, Li SZ. Serum miR-300 as a diagnostic and prognostic biomarker in osteosarcoma. Oncol Lett. 2016; 12:3912–8.
7. Hong Q, Fang J, Pang Y, Zheng J. Prognostic value of the microRNA-29 family in patients with primary osteosarcomas. Med Oncol. 2014; 31:37.
8. Fujiwara T, Uotani K, Yoshida A, Morita T, Nezu Y, Kobayashi E, Yoshida A, Uehara T, Omori T, Sugiu K, Komatsubara T, Takeda K, Kunisada T, et al. Clinical significance of circulating miR-25-3p as a novel diagnostic and prognostic biomarker in osteosarcoma. Oncotarget. 2017; 8:33375–92. https://doi.org/10.18632/oncotarget.16498.
9. Li S, Gao Y, Wang Y, Wang K, Dai ZP, Xu D, Liu W, Li ZL, Zhang ZD, Yang SH, Yang C. Serum microRNA-17 functions as a prognostic biomarker in osteosarcoma. Oncol Lett. 2016; 12:4905–10.
10. Yang Z, Zhang Y, Zhang X, Zhang M, Liu H, Zhang S, Qi B, Sun X. Serum microRNA-221 functions as a potential diagnostic and prognostic marker for patients with osteosarcoma. Biomed Pharmacother. 2015; 75:153–8.
11. Tang J, Zhao H, Cai H, Wu H. Diagnostic and prognostic potentials of microRNA-27a in osteosarcoma. Biomed Pharmacother. 2015; 71:222–6. https://doi.org/10.1016/j.biopha.2015.01.025.
12. Sun Y, He N, Dong Y, Jiang C. MiR-24-BIM-Smac/DIABLO axis controls the sensitivity to doxorubicin treatment in osteosarcoma. Sci Rep. 2016; 6:34238.
13. Ma W, Zhang X, Chai J, Chen P, Ren P, Gong M. Circulating miR-148a is a significant diagnostic and prognostic biomarker for patients with osteosarcoma. Tumour Biol. 2014; 35:12467–72.
14. Fei D, Li Y, Zhao D, Zhao K, Dai L, Gao Z. Serum miR-9 as a prognostic biomarker in patients with osteosarcoma. J Int Med Res. 2014; 42:932–7.
