# Supplementary material for: Circulating biomarkers in osteosarcoma: new translational tools for diagnosis and treatment
Source: Oncotarget. 2017 Aug 3;8(59):100831–51. doi: 10.18632/oncotarget.19852 (PMC5725068; doi:10.18632/oncotarget.19852)
Supplement: Supplementary file 2 [file oncotarget-08-100831-s002.docx]

**Supplementary Table 1:** Clinical significance of OncomiRNA(s) in OS. When no reported in the study, fold increase and decrease in miRNA expression levels between OS pts and HC or between tumour tissue and adjacent normal tissue were calculated as a ratio of reported 2^-ΔΔCt^ (i.e. serum miR-21 2^-ΔΔCt^ in OS / serum miR-21 2^-ΔΔCt^ in HC).

| **OncomiRNA(s)** | **Patient cohort** | **Sample** | **Clinical observation** | **Study** |
| --- | --- | --- | --- | --- |
| miR-21,  miR-143,  miR-199a-3p | 40 OS pts;  40 HC | Plasma  (40 OS & 40 HC) | Higher miR-21 levels (<2-fold increase, p=0.03) and lower miR-143 and miR-199a-3p levels (<2-fold, p<0.05) decrease were found in OS pts compared to HC.  MiR-21 and miR-143 were associated with metastasis status and histological subtype (both p<0.01), while miR-199-3p with histological subtype (p<0.01).  Each of the 3 miRNA differentiated OS from HC: miR-21: AUC=0.863 [95%CI=0.818–0.908]; miR-199a-3p: AUC=0.918 [95%CI=0.882–0.954]; miR-143: AUC=0.902 [95%CI=0.864–0.940].  3-miRNA panel differentiated OS from HC: AUC=0.953 [95%CI=0.924–0.984], sensitivity=0.91; specificity= 0.94. | [1] |
| miR-196a/  miR-196b | 100 OS pts;  100 HC | Serum  (100 OS & 100 HC)  Tissue  (100 tumour tissue &  100 adjacent normal tissue) | Higher miR-196a and miR-196b levels were found in OS pts compared to HC (miR-196a: ≈1.9-fold increase, p<0.001; miR-196b: ≈1.8-fold increase, p<0.001).  Higher miR-196a and miR-196b levels were found in tumour tissue in comparison to adjacent normal one (miR-196a: ≈1.8-fold increase, p<0.001; miR-196b: ≈1.7-fold increase, p<0.001).  Tissue and serum miR-196a and miR-196b levels positively correlated in OS pts (miR-196a: rho=0.62, p=0.01; miR-196b: rho=0.68, p=0.001).  Higher serum miR-196a and miR-196b as well as combined upregulation were associated with high tumour grade (p < 0.01, respectively), metastasis (p< 0.01) and recurrence (p <0.01) of OS pts.  MiR-196a and miR-196b expressions and MiR-196a/miR-196b co-expressions were found to be independent prognostic factors of unfavorable survival (Cox regression) in OS pts for:   - *S* (miR-196a: HR=6.28 [95%CI=1.62–13.39], p=0.01; miR-196b: HR=6.33 [95%CI=1.61–13.48], p=0.01; miR-196a/miR-196b: HR=9.89 [95%CI=2.66–20.98], p=0.001); and - *PFS* (miR-196a: HR=6.95 [95%CI=1.63–14.61], p=0.01; miR-196b: HR=6.98 [95%CI=1.65–14.82], p=0.01; miR-196a/miR-196b: HR=10.09 [95%CI=2.82–21.99], p=0.001). | [2] |
| miR-195-5p, miR-199a-3p, miR-320a,  miR-374a-5p | 90 OS pts;  90 HC | Plasma  (90 OS & 90 HC;  in 50 OS pts, paired pre-surgery &  1-month post-surgery) | Higher miRNAs levels were found in OS pts compared to HC (≥2.0-fold increase, p<0.0001);  4-miRNA panel levels decreased in postoperative samples compared with those in preoperative ones (each p<0.0001).  MiR-195-5p and miR-199a-3p were associated with metastasis status (each p<0.0001), while miR-199a-3p and miR-320a with histological subtype (each p<0.0001).  Each of the 4 miRNA differentiated OS from HC: miR-195-5p: AUC=0.903 [95%CI=0.860–0.946]; miR-199a-3p: AUC=0.903 [95%CI=0.866–0.939]; miR-320a: AUC=0.919 [95%CI=0.886–0.952]; and miR-374a-5p: AUC=0.917 [95%CI=0.8855–0.9492];  4-miRNA panel differentiated OS from HC: AUC=0.908 [95%CI=0.931–0.991], cut-off=3.78, sensitivity=0.91; specificity= 0.94. | [3] |
| miR-199a-5p | 60 OS pts;  60 HC | Serum  (60 OS & 60 HC;  in 28 OS pts, paired pre-surgery &  1-month post-surgery) | Higher miR-199a-5p levels were found in OS pts compared to HC (2.94-fold increase, p<0.0001).  MiR-199-5p differentiated OS from HC: AUC=0.860 [95%CI=0.793–0.928], cut-off=589.2, sensitivity=0.88; specificity= 0.77; (p<0.0001).  MiR-199a-5p levels decreased in postoperative samples compared with those in preoperative ones (p=0.0091). | [4] |
| miR-300 | 20 OS pts | Tissue  (20 tumour tissue &  20 adjacent normal tissue) | Higher miR-300 levels were found in tumour tissue in comparison to adjacent normal one (≈2.2-fold increase, p<0.001). | [5] |
| miR-300 | 114 OS pts  114 HC | Serum  (114 OS – 97 OS treated with curative resection and 17 OS with palliative resection - & 114 HC) | Higher miR-300 levels were found in OS pts compared to HC (**≈**1.3-fold increase, p=0.007).  Tissue and serum miR-300 levels positively correlated in OS pts. (rho=0.25, p=0.0068).  Serum miR-300 differentiated OS from HC: AUC=0.885, cut-off 2.63, sensitivity=0.84; specificity= 0.87.  Higher miR-300 levels were ssociated with advanced clinical stage (p=0.0120) and distant metastasis (p=0.0350).  Serum MiR-300 levels decreased in OS pts following curative surgery (p=0.009).  Serum miR-300 expression was found to be an independent prognostic factor of unfavorable survival (Cox regression) in OS pts for *S* (HR=4.70 [95%CI=1.56–8.37], p=0.014) and *PFS* (HR=4.41 [95%CI=1.29–6.99], p=0.013). | [6] |
| miR-29 family | 80 OS pts;  80 HC | Serum  (80 OS & 80 HC)  Tissues  (80 tumour tissue &  80 adjacent normal tissue) | Higher miR-29a,b,c levels were found in OS pts compared to HC (miR-29a: **≈**3.0-fold increase, p<0.001; miR-29b: **≈**1.6-fold increase, p<0.001; miR-29c: ≈1.4-fold increase, p<0.01).  Higher miR-29a,b,c levels were found in tumour tissue in comparison to adjacent normal one (miR-29a: **≈**1.6-fold increase, p<0.001; miR-29b: **≈**2.0-fold increase, p<0.001; miR-29c: ≈1.2-fold increase, p<0.01).  Higher miR-29a and miR-29b levels were associated with higher tumour grade positive metastasis (both p=0.006) and positive recurrence (P=0.006).  Tissue and serum miR-29a,b,c levels positively correlated in OS pts. (miR-29a: rho=0.303, p=0.007; miR-29b: rho=0.289, p=0.01; miR-29c: rho=0.132, p=0.04).  MiR-29a and miR-29b expressions were found to be independent prognostic factors of unfavorable survival (Cox regression) in OS pts for *S* (miR-29a: HR=5.40 [95%CI=1.22–12.39], p=0.01; miR-29b: HR=5.44 [95%CI=11.31–12.48], p=0.01 ) and *PFS* (miR-29a: HR=5.98 [95%CI=1.23–12.61], p=0.01; miR-29b: HR=6.00 [95%CI=1.35–12.82], p=0.01). | [7] |
| miR-25-3p | 14 OS pts;  14 age-matched non-OS pts;  8 HC | Serum  (14 OS, 14 non-OS & 8 HC) | Higher miR-25-3p levels were found in OS pts compared to HC (**≈**17-fold increase, p=0.004) and non-OS pts (**≈**17-fold increase, p=0.004).  MiR-25-3p differentiated OS from HC: AUC=0.868 [95%CI=0.743–0.993], sensitivity=0.71; specificity= 0.92.  High expression of miR-25-3p was associated (KM compared with L-R) with poor *PFS* (p=0.023). | [8] |
| miR-17 | 46 OS pts;  46 HC | Serum  (46 OS & 46 HC)  Tissues  (46 tumour tissue &  46 adjacent normal tissue) | Higher miR-17 levels were found in OS pts compared to HC (**≈**1.5-fold increase, p=0.025).  Higher tissue miR-17 levels were found in tumour tissue in comparison to adjacent normal one (**≈**2.0-fold increase, p=0.037).  Serum miR-17 levels correlated with tissue phosphatase and tensin homolog (PTEN) expression (r=0.5297, p=0.0002).  High expression of miR-17 was associated (KM compared with L-R) with poor *S* (p=0.0037). | [9] |
| miR-221 | 108 OS pts;  50 HC | Serum  (108 OS & 50 HC)  Tissues  (108 tumour tissue &  108 adjacent normal tissue) | Higher miR-221 levels were found in OS pts compared to HC (1.74-fold increase, p=0.001).  Higher tissue miR-221 levels were found in tumour tissue in comparison to adjacent normal one (2.2-fold increase, p<0.001).  Tissue and serum miR-221 levels positively correlated in OS pts. (rho = 0.506, p<0.001).  MiR-221 differentiated OS from HC: AUC=0.844, cut-off value=1.98, sensitivity=0.66; specificity= 1.00.  MiR-221 expression was found to be an independent prognostic factor of unfavorable survival (Cox regression) in OS pts for *S* (HR=7.66 [95%CI=1.83– 15.92], p=0.01) and *PFS* (HR=6.82 [95%CI=1.33–13.69], p=0.01). | [10] |
| miR-27a | 166 OS pts;  60 HC | Serum  (166 OS & 60 HC) | Higher miR-27a levels were found in OS pts compared to HC (1.85-fold increase, p<0.001).  High miR-27a levels were associated in OS pts with advanced clinical stage (p=0.001), distant metastasis (p=0.01) and poor response to chemotherapy (p=0.008).  MiR-27a differentiated OS from HC: AUC=0.867, cut-off value=2.8, sensitivity=0.70; specificity= 0.98.  MiR-27a expression was found to be an independent prognostic factor of unfavorable survival (Cox regression) in OS pts for *S* (HR=3.56 [95%CI=1.87– 9.86], p=0.01) and *PFS* (HR=3.19 [95%CI=1.72–8.33], p=0.01). | [11] |
| miR-24 | 62 OS pts;  62 HC | Serum  (62 OS & 62 HC)  Tissues  (45 tumour tissue &  45 adjacent normal tissue) | Higher serum miR-24 levels were found in OS pts compared to HC (4.31-fold increase, p<0.05).  Higher tissue miR-24 levels were found in tumour tissue in comparison to adjacent normal one (**≈**6-fold increase, p<0.05). | [12] |
| miR-148a | 89 OS pts;  89 HC | Plasma  (89 OS & 89 HC) | Higher miR-148a levels were found in OS pts compared to HC (0.60-fold increase, p<0.001).  Higher miR-148a levels were associated in OS pts with large tumour size (p=0.049) and distant metastasis (p=0.004).  MiR-148a differentiated OS from HC: AUC=0.783, cut-off value=5.2, sensitivity=0.68; specificity= 0.83.  MiR-148a expression was found to be an independent prognostic factor of unfavorable survival (Cox regression) in OS pts for *S* (95%CI of HR=1.41–5.17, p=0.003) and *PFS* (95%CI of HR=1.28–5.01, p=0.008). | [13] |
| miR-9 | 118 OS pts;  60 HC | Serum  (118 OS & 60 HC) | Higher miR-9 levels were found in OS pts compared to HC (1.76-fold increase, p<0.01).  Higher miR-9 levels were associated in OS pts with advanced TNM stage, large tumour diameter and distant metastasis (p<0.01).  High expression of miR-9 was associated (KM compared with L-R) with poor *S* (p=0.002). | [14] |

Abbreviations: AUC, area under receiving operating characteristics curve; *DFS*, disease free survival; HC, healthy control/volunteers; HR, hazard ratio; KM, Kaplan-Meier survival curve, L-R, log-rank test; OS, osteosarcoma; *PFS*, progression free survival; pts, patients; *S*, overall survival; 95%CI: 95% confidence interval.
